# Construction of non-canonical PAM-targeting adenosine base editors by restriction enzyme-free DNA cloning using CRISPR-Cas9

**DOI:** 10.1038/s41598-019-41356-1

**Published:** 2019-03-20

**Authors:** You Kyeong Jeong, Jihyeon Yu, Sangsu Bae

**Affiliations:** 1 Department of Chemistry, Hanyang University, Seoul, 04763 South Korea; 2 Research Institute for Convergence of Basic Sciences, Hanyang University, Seoul, 04763 South Korea

## Abstract

Molecular cloning is an essential technique in molecular biology and biochemistry, but it is frequently laborious when adequate restriction enzyme recognition sites are absent. Cas9 endonucleases can induce site-specific DNA double-strand breaks at sites homologous to their guide RNAs, rendering an alternative to restriction enzymes. Here, by combining DNA cleavage via a Cas9 endonuclease and DNA ligation via Gibson assembly, we demonstrate a precise and practical DNA cloning method for replacing part of a backbone plasmid. We first replaced a resistance marker gene as a proof of concept and next generated DNA plasmids that encode engineered Cas9 variants (VQR, VRER and SpCas9-NG), which target non-canonical NGA, NGCG and NG protospacer-adjacent motif (PAM) sequences, fused with adenosine deaminases for adenine base editing (named VQR-ABE, VRER-ABE and NG-ABE, respectively). Ultimately, we confirmed that the re-constructed plasmids can successfully convert adenosine to guanine at endogenous target sites containing the non-canonical NGA, NGCG and NG PAMs, expanding the targetable range of the adenine base editing.

## Introduction

Molecular cloning techniques are widely used in molecular biology and biochemistry to construct recombinant DNA molecules. Traditionally, cloning requires appropriate restriction enzyme sites in the DNA insert and plasmid for ligation. However, when researchers wish to manipulate a short region of DNA, only hundreds of base pairs (bps) in length, in a plasmid, appropriate restriction sites near the region of interest are frequently limited; thus, researchers often manipulate a larger DNA fragment instead. Alternatively, polymerase chain reaction (PCR)-based site-directed mutagenesis can be used to engineer DNA sequences in the original plasmid, but such processes require multiple steps to change more than one base pair and can generate sequence errors in unexpected regions^1^.

To date, a few restriction enzyme-independent methods have been developed by using the CRISPR (clustered regularly interspaced short palindromic repeat)-Cas (CRISPR associated) system^2–9^. Type II Cas9 and type V Cpf1 (also called Cas12a) are representative RNA-guided DNA endonucleases that specifically induce DNA double strand breaks at desired sites containing protospacer-adjacent motif (PAM) sequences, 5′-NGG-3′ for SpCas9 derived from *Streptococcus pyogenes* and 5′-TTN-3′ for FnCpf1 from *Francisella novicida*^10,11^. Therefore, researchers can cleave DNA in a region lacking an appropriate enzyme site by using CRISPR endonucleases. Furthermore, it is both inexpensive and easy to change DNA target sites by simply altering the guide RNA sequence. Previously, Jiang *et al*. developed the Cas9-Assisted Targeting of CHromosome segments (CATCH) method^12,13^ and Lei *et al*. developed the Cpf1-assisted Cutting and Taq DNA ligase-assisted Ligation (CCTL) method^14^. CATCH use the CRISPR-Cas system to separate large gene segments and CCTL use the system to induce sticky end; these approaches showed that CRISPR endonucleases can be used to generate greater flexibility in the choice of cleavage sites. These methods adapted the CRISPR system for DNA cleavage and facilitated the introduction of sites into regions that are difficult to modify *via* PCR. On the other hand, Wang *et al*.^15^ developed a seamless DNA cloning method by using CRISPR-Cas9 and Gibson assembly, and showed a simple way to insert new DNA fragment by targeting one site using one guide RNA with Cas9 protein.

In this study, following to the previous methods, we demonstrated a precise DNA cloning method that involves cleaving a part of the backbone plasmid using two kinds of guide RNAs with CRISPR-Cas9 and ligating new DNA fragments using Gibson assembly^16^. Using this method, we altered an antibiotic resistance gene in a DNA plasmid in one step and ultimately constructed plasmids encoding adenine base editors (ABEs)^17,18^, consisting of Cas9 variants that recognize non-canonical PAM sequences, 5′-NGA-3′ for the VQR Cas9 variant, 5′-NGCG-3′ for the VRER Cas9 variant^19,20^, and 5′-NG-3′ for the SpCas9-NG variant^21^, fused with adenosine deaminases. Furthermore, we confirmed the function of the new DNA plasmids (VQR-ABE, VRER-ABE, and NG-ABE) in human cell lines.

## Results and Discussion

### Replacement of selection marker

**Figure 1:**
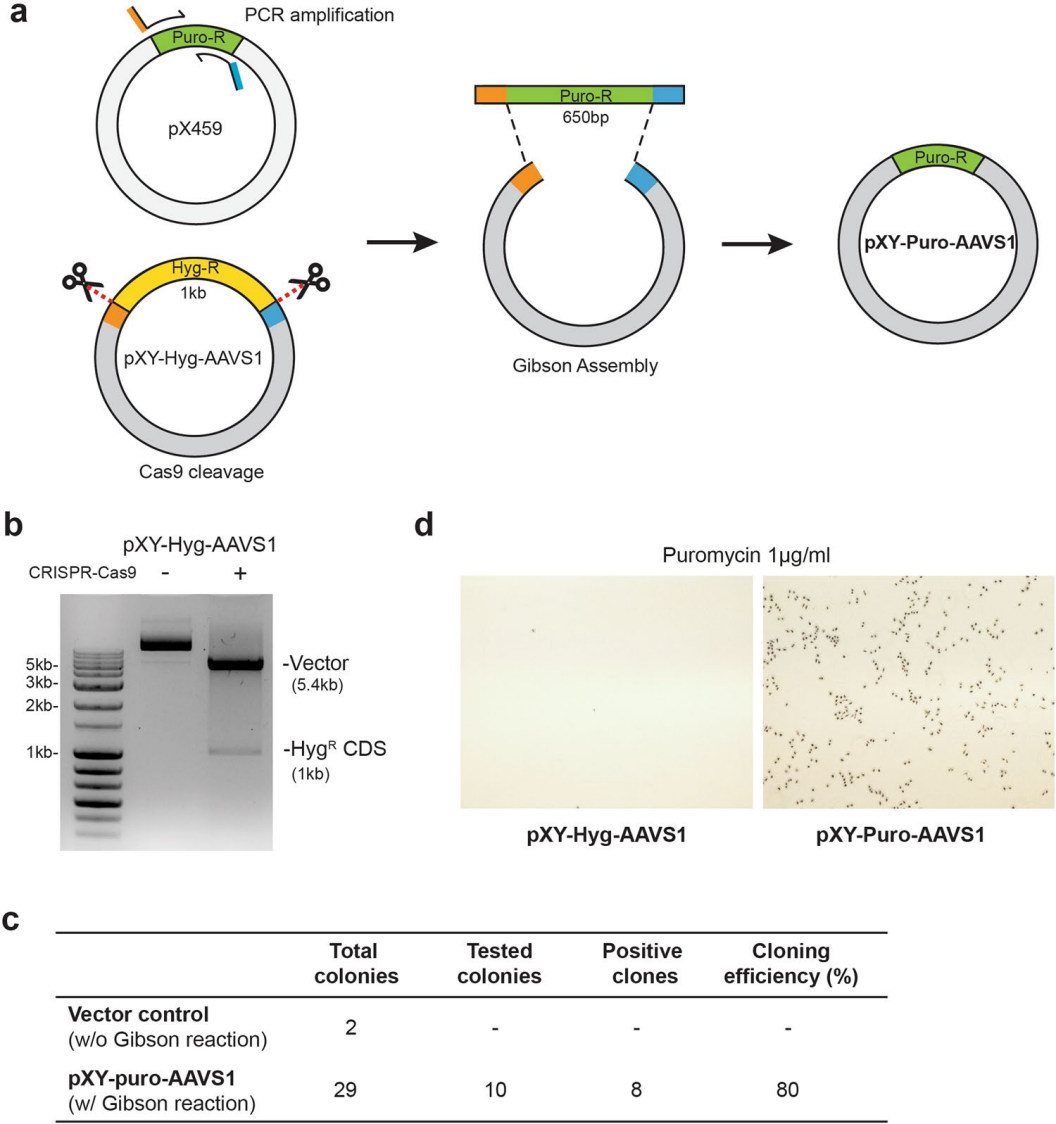
Replacement of antibiotic resistance gene. (**a**) Cloning scheme for exchanging antibiotic resistance genes. Recombinant Cas9 proteins are represented as scissors. Regions that are homologous between the backbone pXY-Hyg-AAVS1 plasmid and the PCR products are indicated in orange and blue. The PCR products and the backbone vector were attached by Gibson assembly. (**b**) Agarose gel image showing DNA cleavage of the backbone plasmid pXY-hyg-AAVS1 via CRISPR-Cas9 for vector preparation. (Lane 1: DNA marker, lane 2: uncut plasmid, lane 3, cut plasmid by CRISPR-Cas9). Full-length gel is presented in Supplementary Fig. 1. (**c**) Summarized table of the cloning efficiency. The linearized vectors were transformed into *E. coli* DH5α competent cells without Gibson assembly reaction as a negative control. The mixtures of linearized vectors and Puro-R amplicons were transformed into the DH5α cells after Gibson reaction. After incubating the DH5α cells in LB plate containing ampicillin, the number of survived colonies were counted on the LB plate (total colonies). Among the ten colonies evaluated by enzyme cutting experiments (tested colonies), eight colonies were successfully constructed (positive colonies). (**d**) Photomicrographs of HeLa cells treated with puromycin (1 μg/ml) after transfection with pXY-Hyg-AAVS1 (left) and pXY-Puro-AAVS1 (right) with 40-fold magnification.

Selection markers such as antibiotic resistance genes are crucial for obtaining user-defined cell lines; it is frequently necessary to exchange an existing selection marker with a desired one. We first demonstrated a precise DNA cloning method by replacing the *hph* (hygromycin B phosphotransferase; Hyg-R) gene with the *pac* (puromycin N-acetyl-transferase; Puro-R) gene in a DNA plasmid. We prepared a pXY-Hyg-AAVS1 vector (6.4kbp) that includes the Hyg-R gene as the backbone plasmid and a PX459 plasmid that includes the Puro-R gene as a donor plasmid (or a PCR template) as shown in Fig. 1a. To cleave the Hyg-R gene from the backbone plasmid *in vitro*, we treated the pXY-Hyg-AAVS1 plasmid with recombinant Cas9 protein complexed with two single-guide RNAs (sgRNAs) that target sites flanking the Hyg-R gene within 100 bp of the Hyg-R gene boundaries in the backbone plasmid^22^ (Fig. 1b and Supplementary Fig. 1). The insert DNA fragments containing the Puro-R gene were amplified from the PX459 plasmid using a pair of PCR primers that contained about 20 bp of additional sequence homologous to sequences flanking Hyg-R in the backbone plasmid. The backbone plasmid lacking the Hyg-R gene and the insert DNA fragments containing Puro-R gene were then mixed and re-ligated *via* the Gibson assembly process. We then transformed the ligated samples into E. coli DH5α competent cells and incubated them in LB plate containing ampicillin. To measure the cloning efficiency, we identified that eight of ten colonies successfully contain Puro-R gene via restriction enzyme cutting experiments (Fig. 1c, Supplementary Fig. 2) and one of eight was further confirmed by Sanger sequencing (Supplementary Fig. 3). As an end result, we obtained a re-constructed backbone plasmid that contained the Puro-R gene, named pXY-Puro-AAVS1. To test whether it would confer puromycin resistance, we separately transfected pXY-Hyg-AAVS1 and pXY-Puro-AAVS1 into HeLa cells and cultivated them in media containing puromycin. As expected, HeLa cells transfected with pXY-Puro-AAVS1 were selectively viable in medium containing 1 μg/ml of puromycin (Fig. 1d), indicating the successful replacement of the selection marker in the backbone plasmid.

### Precise replacement of ABE (7.10) with VQR-ABE and VRER-ABE

Recently, CRISPR base editors that enable direct conversion of DNA bases without generating double-stranded DNA breaks have been developed. Gaudelli *et al*.^17^ constructed an adenine base editor (ABE version 7.10) that consists of deactivated Cas9 (dCas9) or Cas9 nickase (nCas9) linked with wild-type tRNA adenosine deaminase (TadA) – evolved TadA* heterodimer. Because the ABE is based on wild-type (WT) Cas9, it is limited to targeting DNA sequences that contain a 5′-NGG-3′ PAM. On the other hand, Kleinstiver *et al*.^19^ engineered WT Cas9 to expand targetable DNA sequences by altering PAM sequence recognition. The SpCas9 VQR variant (D1135V/R1335Q/T1337R) recognizes ‘NGA’ PAM sequences and the VRER variant (D1135V/G1218R/R1335E/T1337R) recognizes ‘NGCG’ PAM sequences. Compared to WT Cas9, the VQR and VRER variants have 6 and 9 substitutions, respectively, and the total lengths of the variable regions are about 600 bp in each case; thus, DNA fragments in a relatively short region must be exchanged to re-construct VQR-ABE and VRER-ABE.

**Figure 2:**
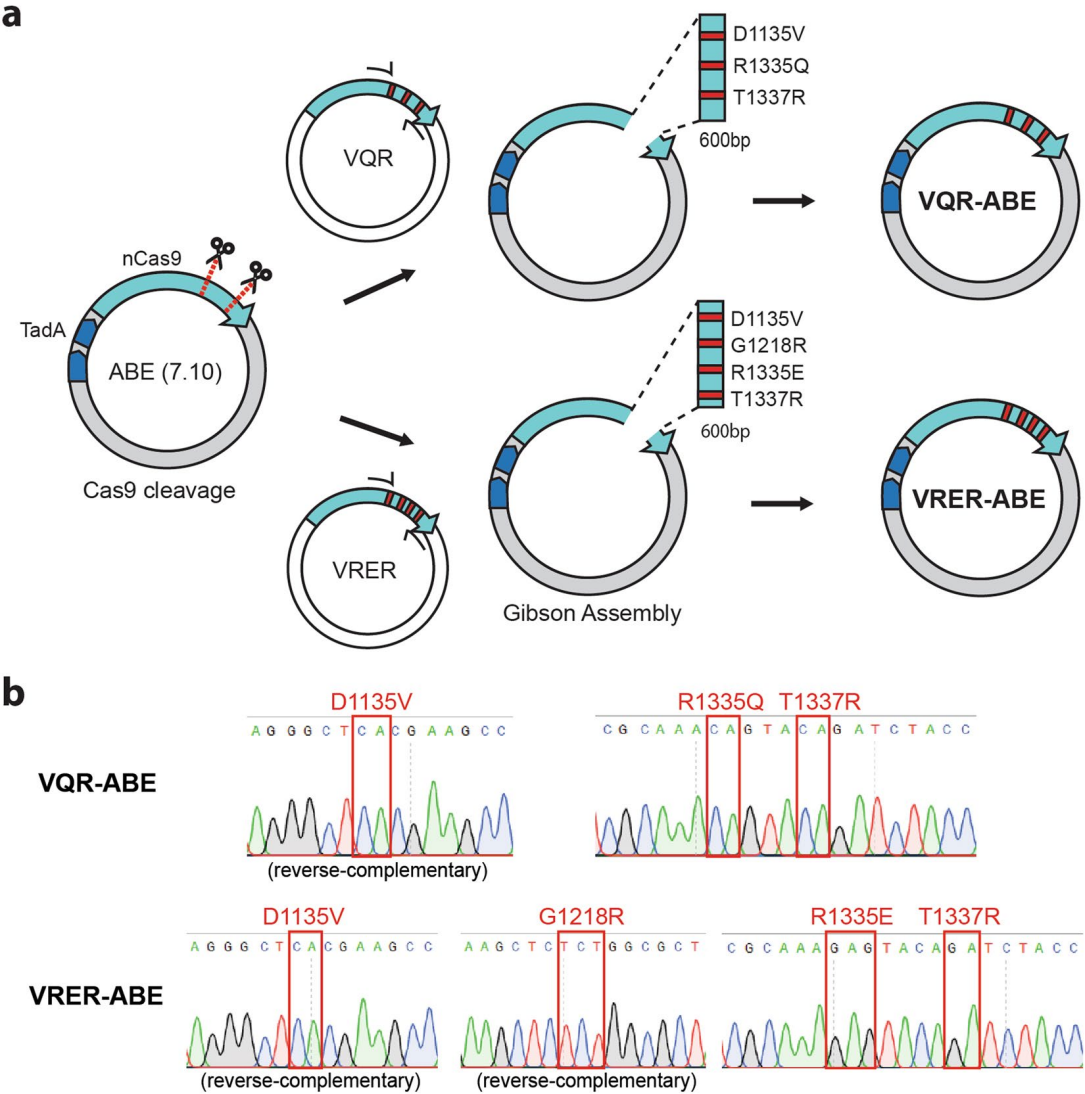
Construction of VQR-ABE and VRER-ABE. (**a**) The WT ABE (7.10) plasmid was prepared as a backbone; VQR and VRER plasmids were used as donors. Deep blue regions encode the WT TadA–evolved TadA* heterodimer. The region to be exchanged in ABE (7.10) was cleaved by CRISPR-Cas9 and the two donor DNA fragments were amplified by PCR. PCR amplicons were attached to the initial backbone vector by Gibson assembly. (**b**) Sanger sequencing data from the regions of interest in the VQR-ABE (top) and VRER-ABE (bottom) plasmids. Red boxes indicate mutations in each plasmid.

Toward this aim, we first prepared the ABE (7.10) vector plasmid encoding WT nCas9 as a backbone plasmid and the two plasmids encoding the VQR or VRER Cas9 variants as donor plasmids (Fig. 2a). We also designed sgRNAs that target just outside of the region to be exchanged in the backbone plasmid. To remove the region of interest from the ABE (7.10) backbone plasmid, we treated it *in vitro* with recombinant Cas9 protein complexed with the sgRNAs. The two insert DNA fragments containing the VQR or VRER Cas9 variants were amplified from the VQR and VRER donor plasmids with primers that also contained about 20 bp of additional sequence homologous to sequences flanking sites of interest in the backbone plasmid for Gibson Assembly. The backbone plasmid lacking the region to be exchanged and the insert DNA fragments containing the VQR or VRER variants were then independently mixed and re-ligated *via* the Gibson assembly process. We ultimately obtained two re-constructed plasmids based on the ABE (7.10) backbone that contain the VQR and VRER variants, named VQR-ABE and VRER-ABE, respectively. We confirmed the substitution of DNA sequences by Sanger sequencing as shown in Fig. 2b.

### Successful gene editing with VQR-ABE and VRER-ABE plasmids in human HEK293T cells

**Figure 3:**
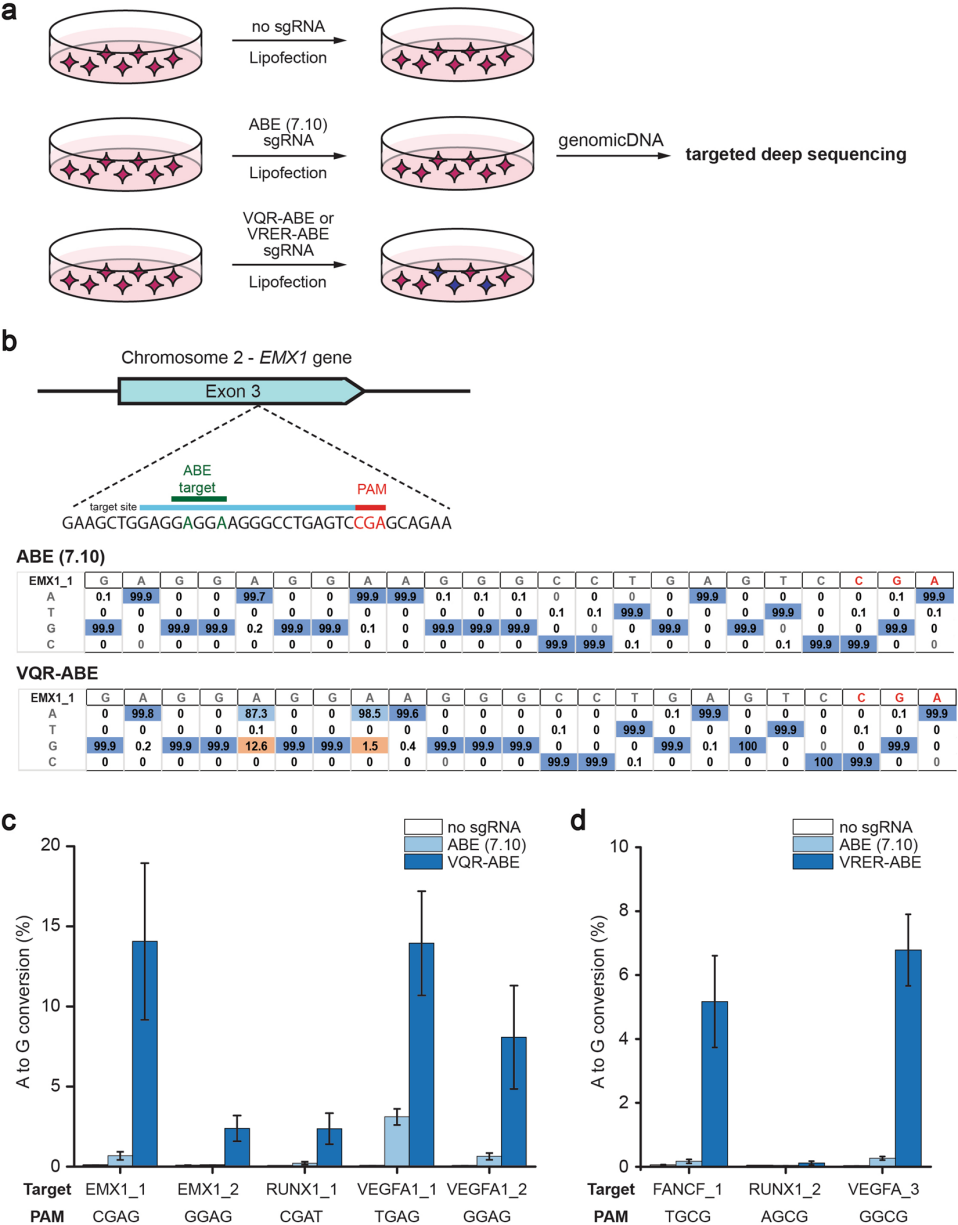
Activity of VQR-ABE and VRER-ABE *in vivo*. (**a**) Human HEK293T cells were transfected with three sets of ABE and sgRNA plasmids. Genomic DNA from treated cells was prepared and analyzed by targeted deep sequencing. (**b**) To test the activity of the VQR-ABE plasmid, one target site containing an ‘NGA’ PAM in the *EMX1* gene was selected (top). The tables show the substitution ratios at each position in the target site after treatment with ABE (7.10) (middle) and VQR-ABE (bottom). The dominant sequence at each position is shown in blue and sequences with a significant level of editing are shown in orange. (**c**,**d**) The graphs show the conversion ratios of A to G at various target sites containing NGA (**c**) or NGCG (**d**) PAMs in human cells for VQR-ABE (**c**) and VRER-ABE (**d**) compared to the negative control and ABE (7.10). All experiments were repeated three times and the error bars in these figures mean the standard error of the mean.

To investigate the adapted breadth of PAM sequences recognized by the ABEs encoded by the VQR-ABE and VRER-ABE plasmids, we examined the base editing efficiency of each plasmid at endogenous DNA site in human cell lines. As shown in Fig. 3a, we transfected the various Cas-ABE plasmids together with sgRNAs into human HEK293T cells and analyzed the base editing efficiencies at the target sites by using targeted deep sequencing. For the VQR-ABE plasmid, we first selected an endogenous target site containing an ‘NGA’ PAM sequence in the *EMX1* gene, which was used in the previous experiment^19^. Targeted deep sequencing data showed that VQR-ABE converted 12.8% of adenines within the target window to guanine, whereas ABE (7.10) was unable to induce significant substitutions (Fig. 3b, Supplementary Table 1)^23,24^. We further tested the VQR-ABE plasmid at another four endogenous target sites. As a result, we found that VQR-ABE converted from 1.1 to 23.1% of adenines within the target windows to guanine, indicating that the VQR-ABE can successfully edit endogenous target sites containing ‘NGA’ PAM sequences, compared to ABE (7.10) and the negative control (Fig. 3c, Supplementary Fig. 4, and Table 1). It is noteworthy that WT ABE (7.10) partially induced A-to-G conversion at target sites having non-canonical ‘NGA’ PAM sequences, similar to the previous study that WT SpCas9 showed low activity on non-canonical ‘NGA’ PAM sequences^20^. Similarly, we tested the base editing efficiency of the VRER-ABE plasmid at endogenous target sites containing ‘NGCG’ PAM sequences. We also confirmed that the VRER-ABE can successfully edit such sequences, compared to ABE (7.10) and the negative control (Fig. 3d, Supplementary Fig. 5, and Supplementary Table 2).

### Construction of NG-ABE plasmids and successful gene editing in human HEK293T cells

**Figure 4:**
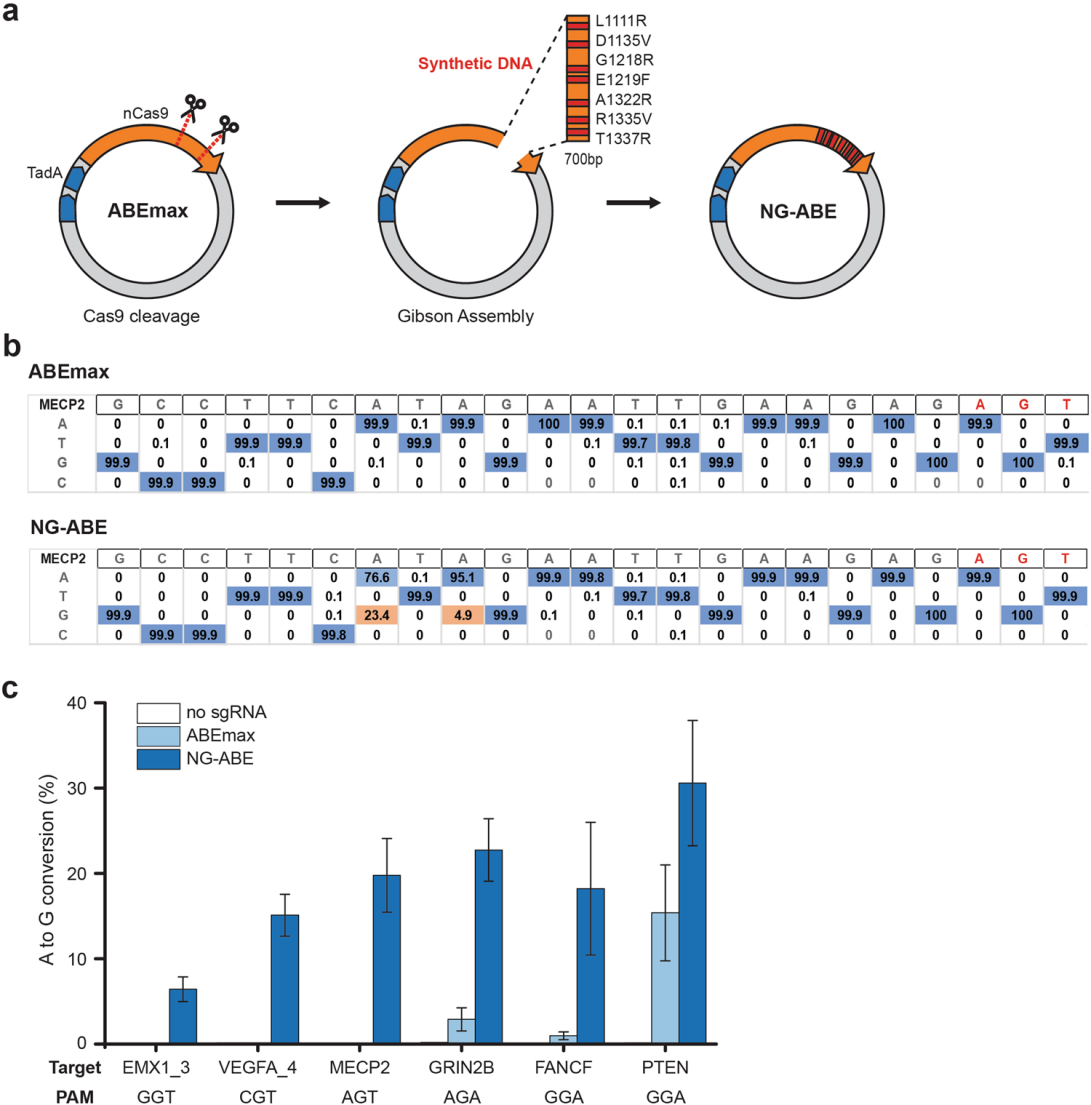
Construction of NG-ABE and its activity test in HEK293T cells. (**a**) The WT ABEmax plasmid was prepared as a backbone and a synthetic DNA that has seven mutations for targeting ‘NG’ PAM was used as an insert DNA. The region to be exchanged in ABEmax was cleaved by CRISPR-Cas9 and the synthetic DNA was attached to the initial backbone vector by Gibson assembly. (**b**) To test the activity of the NG-ABE plasmid *in vivo*, Human HEK293T cells were transfected with NG-ABE and sgRNA plasmids. Genomic DNA from treated cells was prepared and analyzed by targeted deep sequencing. The tables show the substitution ratios at each position in the target site after treatment with ABEmax (top) and NG-ABE (bottom) in one target site containing an ‘NGT’ PAM in the *MECP2* gene. The dominant sequence at each position is shown in blue and sequences with a significant level of editing are shown in orange. (**c**) The graphs show the conversion ratios of A to G at various target sites containing NGT or NGA PAMs in human cells for NG-ABE compared to the negative control and ABEmax. All experiments were repeated three times and the error bars in these figures mean the standard error of the mean.

More recently, Nishimasu *et al*.^21^ developed a new Cas9 variant (R1335V/L1111R/D1135V/G1218R/ E1219F/A1322R/T1337R), named SpCas9-NG, that recognizes a 5′-NG-3′ PAM sequence. To expand the application area of ABEs, we next constructed a ‘NG’ PAM targetable ABE, named NG-ABE. In this case, we prepared a further engineered ABE (7.10) vector plasmid, named ABEmax^25^ as a backbone plasmid and synthetic DNA fragments that have all of seven mutations as insert donors. As described above, we removed the region of interest from the ABEmax backbone plasmid via CRISPR-Cas9 and re-ligated the plasmid with the synthetic insert DNA *via* the Gibson assembly (Fig. 4a).

To evaluate the base editing efficiency of the NG-ABE plasmids at endogenous DNA sites in HEK293T cells, we prepared six kinds of sgRNAs that were designed to target ‘NGT’ or ‘NGA’ PAM sequences, which were used in the previous study^21^. We measured the base editing efficiencies at the target sites by using the targeted deep sequencing. For the target site in the *MECP2* gene, 23.4% of adenines within the target window were converted to guanine via NG-ABE, whereas WT ABEmax showed no significant substitutions as shown in Fig. 4b. In addition, we measured that A-to-G conversions were induced at the ratios of 4.3–32.5% within the target windows for all target sites that we tested, indicating the successful gene editing of NG-ABE on target sites having ‘NG’ PAMs, compared to ABEmax and the negative control (Fig. 4c, Supplementary Fig. 6, and Table 3). We found that ABEmax also induced A-to-G conversion at target sites having non-canonical ‘NGA’ PAM sequences, similar to the above experiments.

In conclusion, we have demonstrated an easy-to-use and restriction enzyme-free DNA cloning method by combining Cas9-mediated DNA cleavage and Gibson assembly. With this method, we replaced an antibiotics resistance marker in a backbone plasmid as a proof of concept and substituted a few base pairs of Cas9 sequence to generate VQR-ABE, VRER-ABE, and NG-ABE plasmids, which can target non-canonical NGA, NGCG, and NG PAMs, in one step. Compared to site-directed mutagenesis, this method avoids unpredictable errors in the vector plasmid caused by repeated PCR and is not limited by the length of the DNA region that will be changed. Although this method needs PAM sequences near DNA cleavage sites in the backbone plasmid, it undoubtedly expands the cleavage sites compared to the restriction enzymes. Given its precise and practical nature, we expect that this method will be useful to a broad range of researchers.

## Materials and Methods

### *In vitro* transcription of single-guide RNAs

Forward oligo sequences, containing the T7 RNA polymerase promoter and the 20 bp target sequence, and a common reverse oligo sequence, containing the guide RNA scaffold region, were synthesized by Macrogen. The two oligo sequences were extended by phusion polymerase (Thermo Fisher Scientific, Thermo Scientific^TM^, catalog number: F530L) in a mixture containing 5X Phusion HF Buffer (F-518L, F-538L (Phusion Green), F-520L (Detergent-free)), 10 mM dNTPs, and distilled water. After template extension, the resulting DNAs were purified using an MG^TM^PCR sv kit (MGmed). The template DNAs were transcribed by T7 RNA polymerase (New England Biolabs, catalog number: M0251L) in a mixture containing 100 mM MgCl_2_, 100 nM NTPs, 10x T7 RNA buffer (New England Biolabs), DEPC-treated water, and 100U RNase inhibitor (New England Biolabs, catalog number: M0314L). After overnight incubation at 37 °C, DNA templates were destroyed with DNase I (RNase-free) (New England Biolabs, catalog number: M0303L) and the RNA products were purified with an RNeasy MinElute Cleanup Kit (QIAGEN, catalog number: 74204).

### Expression and purification of Cas9 protein

Plasmid pET28a-SpCas9, for recombinant SpCas9 protein expression in *E*. *coli*, was provided by Prof. Jin-Soo Kim. Competant BL21-Pro cells (CP111, Ezynomics), transformed with pET28a-SpCas9, were grown to mid-log phase (OD_600_ nm ~0.4–0.5) in LB medium containing kanamycin at 37 °C with shaking at 200 rpm. Protein expression was induced with the addition of 0.8 mM IPTG, after which cells were incubated overnight at 18 °C with shaking at 200 rpm. Cell pellets were obtained by centrifugation at 4 °C (3,100 × g, 20 min). After cells were resuspended in lysis buffer (50 mM NaH_2_PO_4_, 300 mM NaCl, 10 mM imidazole), 2 mg/ml lysozyme and 1 mM PMSF were added. The lysate was incubated for 1 hour on ice and sonicated (amplitude 25%, 1 s ON/1 s OFF, pulse 30 times) eight times. The lysate was then cleared by centrifugation at 4 °C (18,000 × g, 30 min). The supernatant was filtered through a 0.45-um syringe filter and then incubated with Ni-NTA agarose for 1 h with rotation at 4 °C. The resin was then washed twice with wash buffer (50 mM NaH_2_PO_4_, 300 mM NaCl, 20 mM imidazole). The bound proteins were eluted with elution buffer (50 mM NaH_2_PO_4_, 300 mM NaCl, 250 mM imidazole). The eluted proteins were diluted with storage buffer (150 mM NaCl, 20 mM HEPES, 0.1 mM EDTA, 1 mM DTT, 2% sucrose, 20% glycerol). The concentration of the purified protein was determined on an SDS-PAGE gel (Supplementary Fig. 7).

### *In vitro* DNA cleavage using CRISPR-Cas9

1 μg of SpCas9 protein and 0.35 μg of both sgRNA1 and sgRNA2 were pre-incubated for 5 minutes at room temperature, after which 1ug of pXY-Hyg-AAVS1 plasmid or ABE (7.10) plasmid, 3.1 NEB buffer, and DEPC water were added to the SpCas9–sgRNA complexes to the final volume 50 ul. The mixture was incubated at 37 °C for 4 hours to allow cleavage to take place. Products were mixed with loading dye containing SDS and analyzed on a 1.5% agarose gel.

### Gibson assembly

The cleaved backbone plasmid were prepared by *in vitro* cleavage using CRISPR-Cas9 and appropriate PCR product were amplified using PCR templates; pSpCas9(BB)-2A-Puro (PX459) was a gift from Feng Zhang (Addgene plasmid # 48139; http://n2t.net/addgene:48139; RRID:Addgene_48139), MSP469 was a gift from Keith Joung (Addgene plasmid # 65771; http://n2t.net/addgene:65771; RRID:Addgene_65771) and MSP1101 was a gift from Keith Joung (Addgene plasmid # 65773; http://n2t.net/addgene:65773; RRID:Addgene_65773). The backbone plasmid and insert PCR product mixed in a volume of 10 μl containing 2U T5 exonuclease (New England Biolabs, catalog number: M0363S), 12.5U phusion DNA polymerase (Thermo Fisher Scientific, Thermo Scientific^TM^, catalog number: F530L), 2kU Taq DNA ligase (New England Biolabs, catalog number: M0208S), 0.2 M Tris-HCl (pH 7.5), 0.2 M MgCl_2_, 2 mM dNTP, 0.2 M dithiothreitol, PEG 8000, and 1 mM NAD and incubated at 50 °C for 1 hour. The products were then transformed into 100 μl of DH5α competent cells. Single transformed colonies were inoculated into LB medium containing antibiotics. DNA products were isolated from cells using a DNA prep kit (MGmed).

### Restriction enzyme cutting experiment

300 ng of Plasmid DNA was incubated with 1 unit of KpnI (New England Biolabs, catalog number: R31452S) and StuI (Enzynomic, catalog number: R025S) at 37 °C for 1 hour. Cleavage products were loaded and analyzed on 1.2% agarose gel.

### Transfection of pXY-Hyg-AAVS1 and pXY-Puro-AAVS1 plasmids into HeLa cells and of ABE plasmids into HEK293T cells

Both HeLa cells (ATCC^®^ CCL-2^™^) and HEK293T cells (ATCC^®^ CRL-3216™) were cultivated in DMEM medium and the cell density was estimated with a hemocytometer and microscope. 1 × 10^5^ of both cells per well were cultivated in 24-well plates over-night. 100 μl of Opti-MEM medium and 2 μl of lipofectamine 2000 were added to 1 μg of plasmid (pXY-Hyg-AAVS1 or pXY-Puro-AAVS1) or both 750 ng of ABE plasmid and 250 ng of sgRNA plasmid. Each mixture was incubated for 20 minutes at room temperature. After incubation, each mixture was added drop-wise to the corresponding cells. After 3 days, HEK293T cells were detached with 0.05% Trypsin-EDTA. And 1 μg/ml of puromycin was added to the cultivated HeLa cell medium. After puromycin was added, HeLa cells were treated by trypan blue (Sigma-Aldrich, catalog number: T8154). Images were captured after 5-minute incubation by a microscope with 40- and 100- fold magnifications.

### Targeted deep sequencing

HEK293T cells which were cultivated in 3 days were pelleted by centrifugation. The cell pellet was mixed with 100 μl of Proteinase K extraction buffer [40 mM Tris-HCl (pH 8.0), 1% Tween-20, 0.2 mM EDTA, 10 mg of proteinase K, 0.2% nonidet P-40] and incubated at 60 °C for 15 minutes followed by an incubation at 98 °C for 5 minutes. Genomic DNAs of the HEK293T cells were amplified using SUN-PCR blend (SUN GENETICS) and the resulting PCR products were analyzed using an Illumina Mini-Seq instrument. The results of Mini-seq were analyzed using BE-Analyzer (http://www.rgenome.net/be-analyzer/)^23^. PCR primers used in this study are shown in Supplementary Table 4.

## Supplementary information

Supplementary Information
