# Supplementary material for: Construction of non-canonical PAM-targeting adenosine base editors by restriction enzyme-free DNA cloning using CRISPR-Cas9
Source: Sci Rep. 2019 Mar 20;9:4939. doi: 10.1038/s41598-019-41356-1 (PMC6426851; doi:10.1038/s41598-019-41356-1)
Supplement: Supplementary file 1 — Supplementary Information [file 41598_2019_41356_MOESM1_ESM.docx]

**Supplementary Information**

**Construction of non-canonical PAM-targeting adenosine base editors by restriction enzyme-free DNA cloning using CRISPR-Cas9**

You Kyeong Jeong, Jihyeon Yu, and Sangsu Bae

**Content:**

**Supplementary Figure 1.** Raw image of agarose gel in Figure 1b

**Supplementary Figure 2.** Cloning efficiency test done by a restriction enzyme cutting experiment

**Supplementary Figure 3.** Sanger sequencing data from Puro-R region of pXY-Puro-AAVS1 plasmid

**Supplementary Figure 4.** The tables showing the substitution ratios at each position in each target sites after treatment with WT ABE (7.10) and VQR-ABE in turn

**Supplementary Figure 5.** The tables showing the substitution ratios at each position in each target sites after treatment with WT ABE (7.10) and VRER-ABE in turn

**Supplementary Figure 6.** The tables showing the substitution ratios at each position in each target sites after treatment with WT ABEmax and NG-ABE in turn

**Supplementary Figure 7.** Purification of recombinant SpCas9 protein

**Supplementary Table 1.** Targeted deep sequencing data analyzed by BE-Analyzer at various target sites containing NGA PAMs in human cells of negative control, WT ABE (7.10), and VQR-ABE

**Supplementary Table 2.** Targeted deep sequencing data analyzed by BE-Analyzer at various target sites containing NGCG PAMs in human cells of negative control, WT ABE (7.10), and VRER-ABE

**Supplementary Table 3.** Targeted deep sequencing data analyzed by BE-Analyzer at various target sites containing NG PAMs in human cells of negative control, ABEmax, and NG-ABE **Supplementary Table 4.** PCR primers used in this study

**Supplementary Figure 1.** Raw image of agarose gel in Figure 1b.


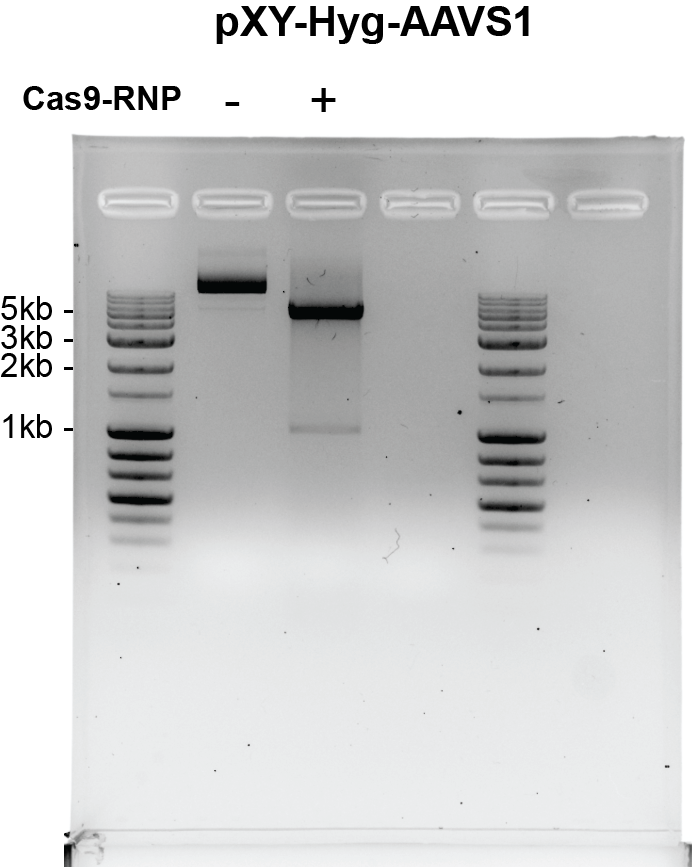


**Supplementary Figure 2.** Cloning efficiency test done by a restriction enzyme cutting experiment. The re-constructed backbone plasmid containing Puro-R gene (pXY-puro-AAVS1) was treated with two restriction enzymes, KpnI and StuI. KpnI recognition site was located at the initial vector (pXY-Hyg-AAVS1) and StuI recognition site was located at the Puro-R gene region. Positive clones showed two bands (4.5kb and 1.4kb) in agarose gel (black arrows), whereas the control (pXY-Hyg-AAVS1) and negative clones (not re-constructed) showed just one band (5.9kb).


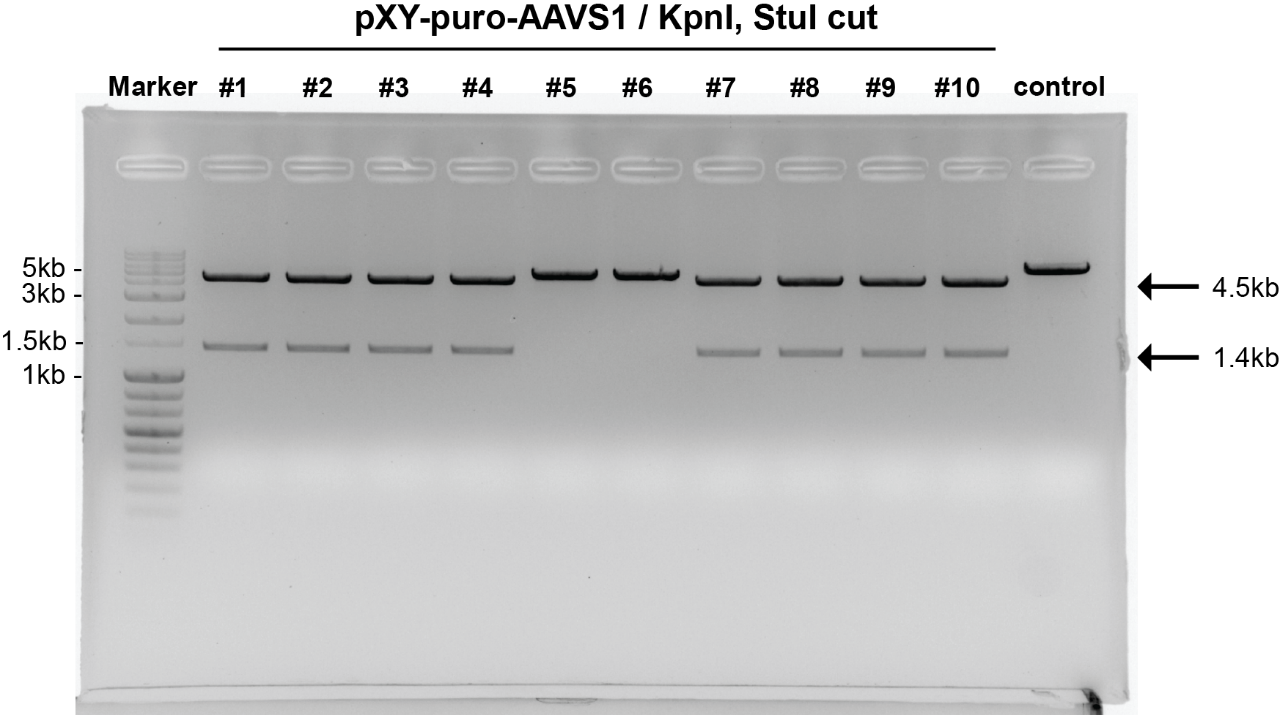


**Supplementary Figure 3.** Sanger sequencing data from Puro-R region of pXY-Puro-AAVS1 plasmid. Sanger sequencing started from HSV-TKp-F primer site which is near Puro-R gene in pXY-Puro-AAVS1 plasmid. Whole sequences in the figure identify original Puro-R gene.

**
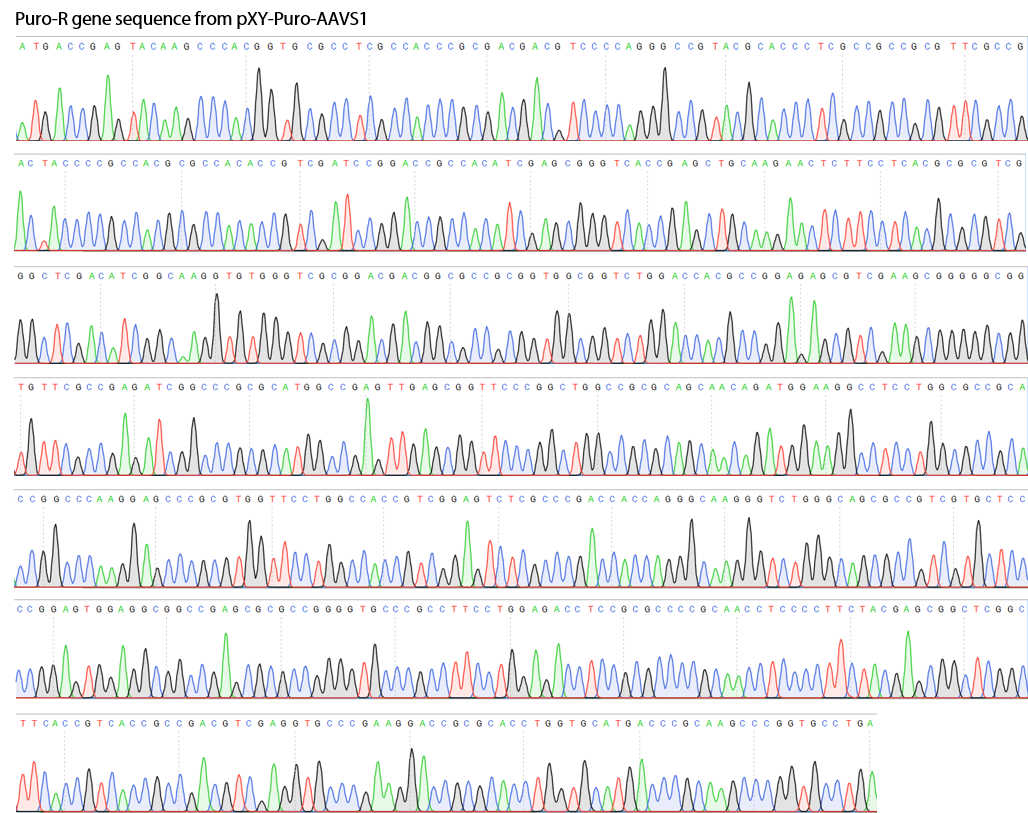
**

**Supplementary Figure 4.** The tables showing the substitution ratios at each position in each target sites after treatment with WT ABE (7.10) and VQR-ABE in turn. The tables show three independent sequencing results performed on different days with HEK293T cells. The dominant sequence at each position is shown in blue and sequences with a significant level of editing are shown in orange.


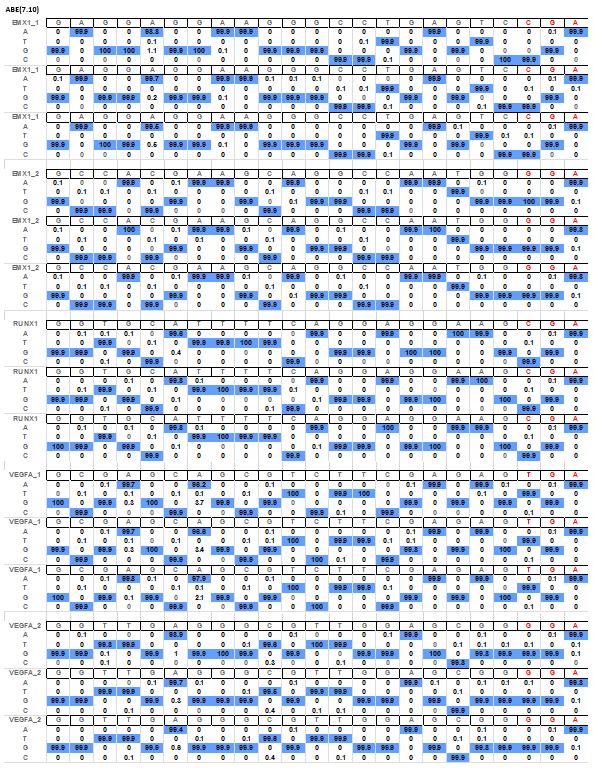


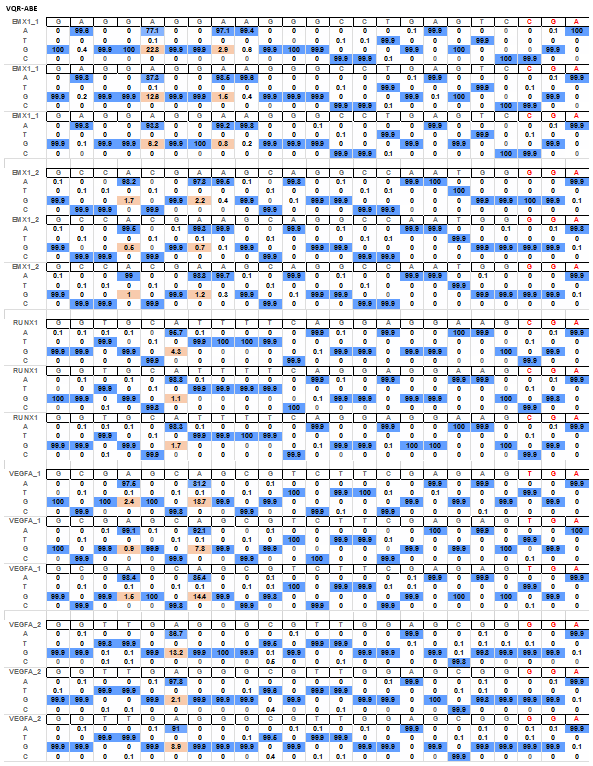


**Supplementary Figure 5.** The tables showing the substitution ratios at each position in each target sites after treatment with WT ABE (7.10) and VRER-ABE in turn. The tables show three independent sequencing results performed on different days with HEK293T cells. The dominant sequence at each position is shown in blue and sequences with a significant level of editing are shown in orange.


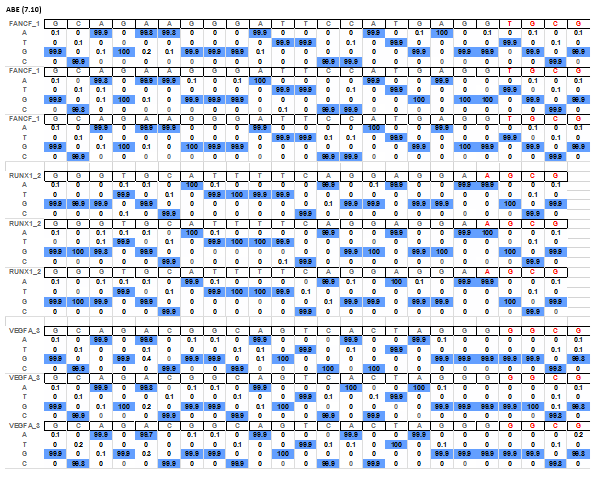


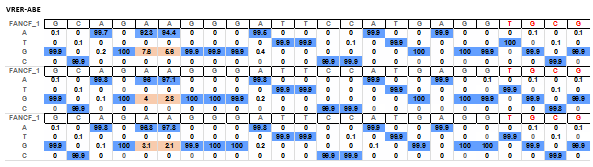


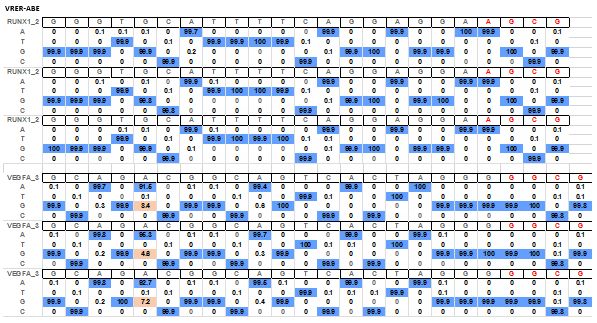


**Supplementary Figure 6.** The tables showing the substitution ratios at each position in each target sites after treatment with WT ABEmax and NG-ABE in turn. The tables show three independent sequencing results performed on different days with HEK293T cells. The dominant sequence at each position is shown in blue and sequences with a significant level of editing are shown in orange.


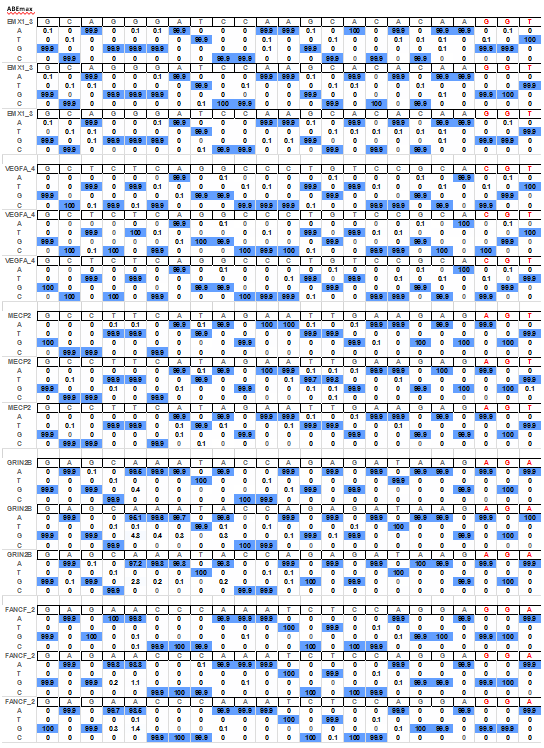


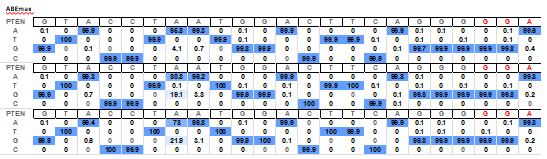


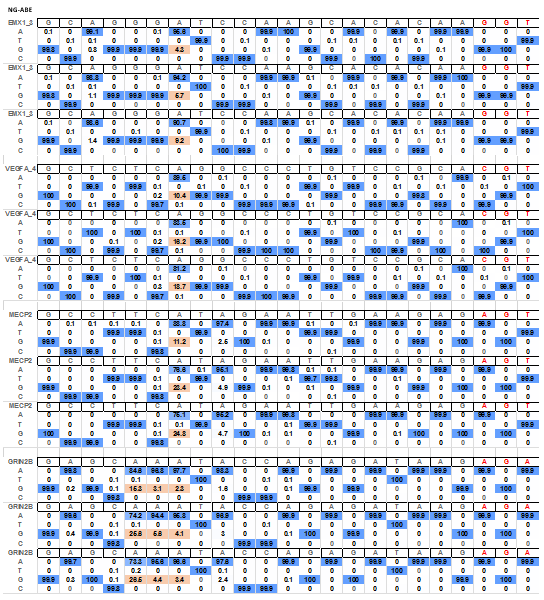


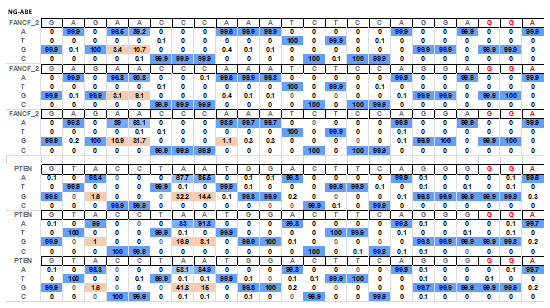


**Supplementary Figure 7.** Purification of recombinant SpCas9 protein. (a) SDS-PAGE (sodium dodecyl sulfate polyacrylamide gel electrophoresis) gel image shows the enrichment of Cas9 protein by Ni-NTA elution. Bottom numbers indicate the defined concentration of BSA (bovine serum albumin). (b) Full-length SDS-PAGE gel of (a). The image in red square was cropped as shown in (a). Outside of the box indicated marker and other enzymes that are unrelated to the manuscript.

**
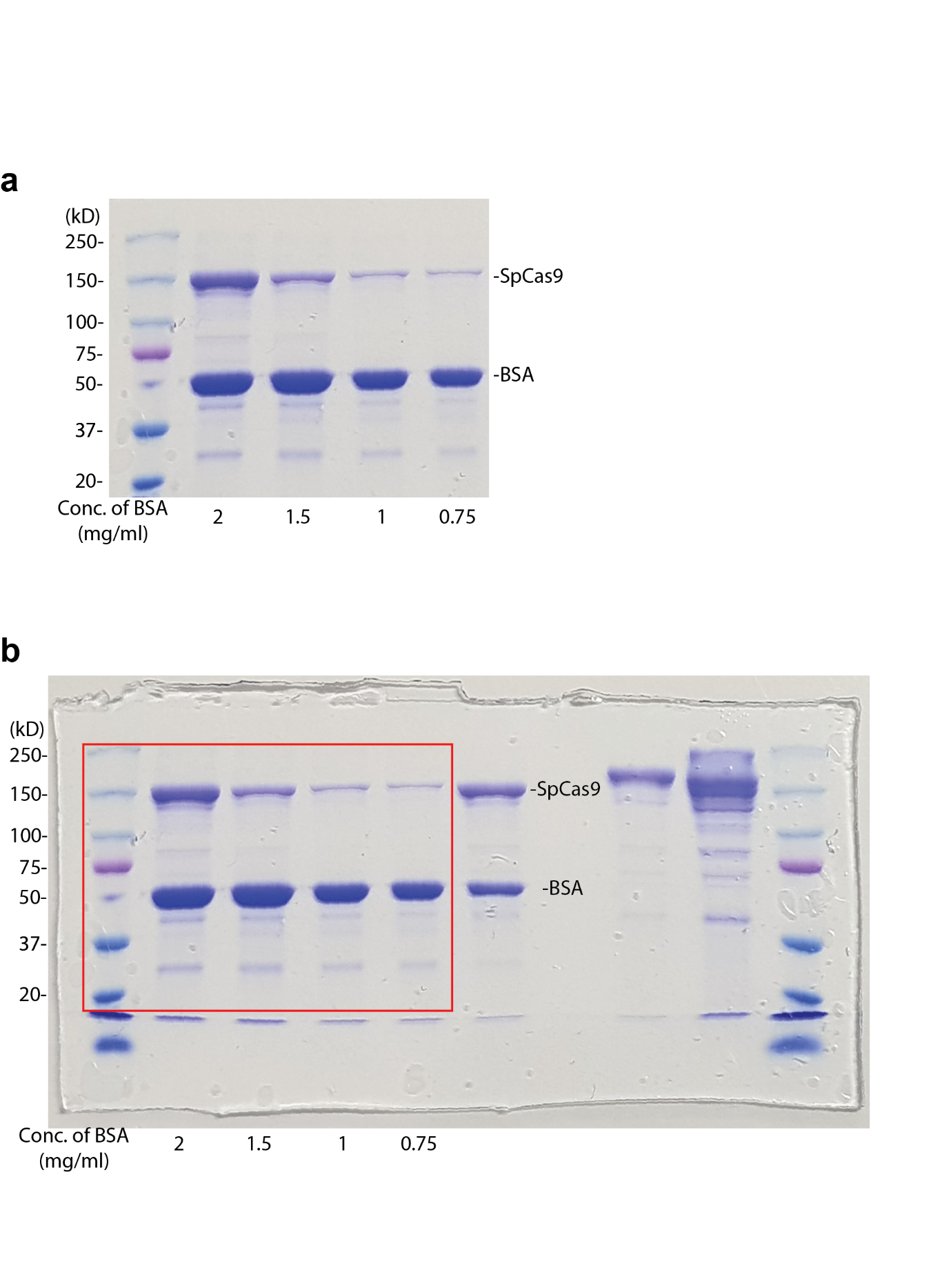
**

**Supplementary Table 1. Targeted deep sequencing data analyzed by BE-Analyzer at various target sites containing NGA PAMs in human cells of negative control, WT ABE (7.10), and VQR-ABE.**

| **Endogenous target** | **Target DNA seq (w/PAM)** | **Total Counts** | | | **A to G substitution Ratios (%)** | | |
| --- | --- | --- | --- | --- | --- | --- | --- |
|  |  | 1st trial | 2nd trial | 3rd trial | 1st trial | 2nd trial | 3rd trial |
| Ctl_EMX1_1 | GAGGAGGAAGGGCCTGAGTCCGAG | 24014 | 28291 | 42971 | 0.07 | 0.08 | 0.1 |
| Ctl_EMX1_2 | GCCACGAAGCAGGCCAATGGGGAG | 34610 | 34870 | 57905 | 0.09 | 0.08 | 0.1 |
| Ctl_RUNX1_1 | GGTGCATTTTCAGGAGGAAGCGAT | 31526 | 54746 | 57467 | 0.02 | 0.03 | 0.04 |
| Ctl_VEGFA_1 | GCGAGCAGCGTCTTCGAGAGTGAG | 24061 | 26906 | 46954 | 0.03 | 0.03 | 0.06 |
| Ctl_VEGFA_2 | GGTTGAGGGCGTTGGAGCGGGGAG | 25413 | 38109 | 52308 | 0.03 | 0.05 | 0.02 |
| ABE(7.10)_EMX1_1 | GAGGAGGAAGGGCCTGAGTCCGAG | 28948 | 33201 | 43870 | 1.16 | 0.3 | 0.56 |
| ABE(7.10)_EMX1_2 | GCCACGAAGCAGGCCAATGGGGAG | 37573 | 47484 | 55710 | 0.1 | 0.08 | 0.06 |
| ABE(7.10)_RUNX1_1 | GGTGCATTTTCAGGAGGAAGCGAT | 35632 | 49137 | 49178 | 0.39 | 0.12 | 0.14 |
| ABE(7.10)_VEGFA_1 | GCGAGCAGCGTCTTCGAGAGTGAG | 25835 | 38562 | 38261 | 3.77 | 3.44 | 2.12 |
| ABE(7.10)_VEGFA_2 | GGTTGAGGGCGTTGGAGCGGGGAG | 25561 | 37637 | 47398 | 1.04 | 0.32 | 0.56 |
| VQR-ABE_EMX1_1 | GAGGAGGAAGGGCCTGAGTCCGAG | 26391 | 46417 | 43795 | 23.08 | 6.28 | 12.81 |
| VQR-ABE_EMX1_2 | GCCACGAAGCAGGCCAATGGGGAG | 34369 | 43248 | 55079 | 3.86 | 1.09 | 2.22 |
| VQR-ABE_RUNX1_1 | GGTGCATTTTCAGGAGGAAGCGAT | 33433 | 31720 | 40341 | 4.27 | 1.12 | 1.71 |
| VQR-ABE_VEGFA_1 | GCGAGCAGCGTCTTCGAGAGTGAG | 44760 | 23965 | 33470 | 19.14 | 7.95 | 14.74 |
| VQR-ABE_VEGFA_2 | GGTTGAGGGCGTTGGAGCGGGGAG | 34779 | 29061 | 39135 | 13.2 | 2.11 | 8.92 |

**Supplementary Table 2. Targeted deep sequencing data analyzed by BE-Analyzer at various target sites containing NGCG PAMs in human cells of negative control, WT ABE (7.10), and VRER-ABE.**

| **Endogenous target** | **Target DNA seq (w/PAM)** | **Total Counts** | | | **A to G substitution Ratios (%)** | | |
| --- | --- | --- | --- | --- | --- | --- | --- |
|  |  | 1st trial | 2nd trial | 3rd trial | 1st trial | 2nd trial | 3rd trial |
| Ctl_FANCF_1 | GCAGAAGGGATTCCATGAGGTGCG | 34492 | 59973 | 72015 | 0.04 | 0.06 | 0.07 |
| Ctl_RUNX1_2 | GGGTGCATTTTCAGGAGGAAGCG | 31526 | 54746 | 57467 | 0.02 | 0.03 | 0.04 |
| Ctl_VEGFA_3 | GCAGACGGCAGTCACTAGGGGGCG | 29398 | 40129 | 55655 | 0.03 | 0.01 | 0.01 |
| ABE(7.10)_FANCF_1 | GCAGAAGGGATTCCATGAGGTGCG | 55377 | 36986 | 53026 | 0.29 | 0.1 | 0.12 |
| ABE(7.10)_RUNX1_2 | GGGTGCATTTTCAGGAGGAAGCG | 49212 | 31835 | 39612 | 0.02 | 0.02 | 0.03 |
| ABE(7.10)_VEGFA_3 | GCAGACGGCAGTCACTAGGGGGCG | 42625 | 30756 | 46319 | 0.35 | 0.16 | 0.29 |
| VRER-ABE_FANCF_1 | GCAGAAGGGATTCCATGAGGTGCG | 48236 | 34069 | 51087 | 7.99 | 4.22 | 3.3 |
| VRER-ABE_RUNX1_2 | GGGTGCATTTTCAGGAGGAAGCG | 46120 | 29731 | 39784 | 0.23 | 0.04 | 0.08 |
| VRER-ABE_VEGFA_3 | GCAGACGGCAGTCACTAGGGGGCG | 43816 | 26577 | 45003 | 8.44 | 4.65 | 7.25 |

**Supplementary Table 3. Targeted deep sequencing data analyzed by BE-Analyzer at various target sites containing NG PAMs in human cells of negative control, WT ABEmax, and NG-ABE.**

| **Endogenous target** | **Target DNA seq (w/PAM)** | **Total Counts** | | | **A to G substitution Ratios (%)** | | |
| --- | --- | --- | --- | --- | --- | --- | --- |
|  |  | 1st trial | 2nd trial | 3rd trial | 1st trial | 2nd trial | 3rd trial |
| Ctl_EMX1_3 | GCAGGGATCCAAGCACACAAGGT | 48861 | 54543 | 32135 | 0.02 | 0.03 | 0.04 |
| Ctl_VEGFA_4 | GCTCTCAGGCCCTGTCCGCACGT | 46268 | 14612 | 31342 | 0.05 | 0.06 | 0.09 |
| Ctl_MECP2 | GCCTTCATAGAATTGAAGAGAGT | 54646 | 67409 | 50140 | 0.03 | 0.06 | 0.06 |
| Ctl_GRIN2B | GAGCAAATACCAGAGATAAGAGA | 58274 | 61852 | 45581 | 0.09 | 0.11 | 0.11 |
| Ctl_FANCF_2 | GAGAACCCAAATCTCCAGGAGGA | 56377 | 61839 | 53343 | 0.06 | 0.04 | 0.06 |
| Ctl_PTEN | GTACCTAATGGACTTCAGGGGGA | 52693 | 42612 | 26057 | 0.05 | 0.08 | 0.06 |
| ABEmax_EMX1_3 | GCAGGGATCCAAGCACACAAGGT | 48741 | 45251 | 38122 | 0.02 | 0.03 | 0.03 |
| ABEmax_VEGFA_4 | GCTCTCAGGCCCTGTCCGCACGT | 47691 | 11729 | 31892 | 0.07 | 0.1 | 0.04 |
| ABEmax_MECP2 | GCCTTCATAGAATTGAAGAGAGT | 58306 | 51792 | 50641 | 0.02 | 0.07 | 0.05 |
| ABEmax_GRIN2B | GAGCAAATACCAGAGATAAGAGA | 64362 | 49394 | 50462 | 0.48 | 5.2 | 3 |
| ABEmax_FANCF_2 | GAGAACCCAAATCTCCAGGAGGA | 77842 | 54386 | 51827 | 0.09 | 1.24 | 1.6 |
| ABEmax_PTEN | GTACCTAATGGACTTCAGGGGGA | 72823 | 40119 | 24264 | 4.27 | 19.39 | 22.48 |
| NG-ABE_EMX1_3 | GCAGGGATCCAAGCACACAAGGT | 65734 | 43892 | 35108 | 4.35 | 5.71 | 9.22 |
| NG-ABE_VEGFA_4 | GCTCTCAGGCCCTGTCCGCACGT | 56313 | 11812 | 30039 | 10.41 | 16.25 | 18.67 |
| NG-ABE_MECP2 | GCCTTCATAGAATTGAAGAGAGT | 86617 | 51855 | 58935 | 11.18 | 23.39 | 24.78 |
| NG-ABE_GRIN2B | GAGCAAATACCAGAGATAAGAGA | 81922 | 49934 | 46806 | 15.46 | 25.96 | 26.82 |
| NG-ABE_FANCF_2 | GAGAACCCAAATCTCCAGGAGGA | 95387 | 47339 | 49468 | 11.29 | 9.61 | 33.75 |
| NG-ABE_PTEN | GTACCTAATGGACTTCAGGGGGA | 79002 | 36482 | 27470 | 32.54 | 17 | 42.2 |

**Supplementary Table 4. PCR primers used in this study.**

| **Primer** | **Sequence (5'-to-3')** |
| --- | --- |
| IVT_common_R | aaaaaagcaccgactcggtgccactttttcaagttgataacggactagccttattttaacttgctatttctagctctaaaac |
| Hyg_sg1_IVT_F | gaaattaatacgactcactatag TTTCATATCTCATTGCCCCC gttttagagctagaaatagcaag |
| Hyg_sg2_IVT_F | gaaattaatacgactcactatag GGGGGAGGCTAACTGAAACA gttttagagctagaaatagcaag |
| Puro_F | gcgtgccgcagatcccggggggcaaGCCGCCATGACCGAGTACAAGCCCACGGTGC |
| Puro_R | ggtattgtctccttccgtgtTCAGGCACCGGGCTTGCGGGTCATG |
| ABE_gRNA1_IVT_F | gaaattaatacgactcactatag TGGGACCCGAAAAAGTACGG gttttagagctagaaatagcaag |
| ABE_gRNA2_IVT_F | gaaattaatacgactcactatag TGTCGCGTCTAGCACCTCCT gttttagagctagaaatagcaag |
| VQR_VRER_F | aaaaggactgggacccgaaa |
| VQR_VRER_R | tgaatcagtgtcgcgtctag |
| EMX1_1stF | ccatccccttctgtgaatgt |
| EMX1_1stR | aatctaccaccccaggctct |
| EMX1_1_2ndF | ACACTCTTTCCCTACACGAC GCTCTTCCGATCT ggcctcctgagtttctcatct |
| EMX1_1_2ndR | GTGACTGGAGTTCAGACGTGT GCTCTTCCGATCT gttgcccaccctagtcattg |
| EMX1_2_2ndF | ACACTCTTTCCCTACACGAC GCTCTTCCGATCT ggacaaagtacaaacggcaga |
| EMX1_2_2ndR | GTGACTGGAGTTCAGACGTGT GCTCTTCCGATCT agtggccagagtccagctt |
| FANCF_1stF | ccggaaattctcggtaggat |
| FANCF_1stR | aagttcgctaatcccggaac |
| FANCF_2ndF | ACACTCTTTCCCTACACGAC GCTCTTCCGATCT agcattgcagagaggcgtat |
| FANCF_2ndR | GTGACTGGAGTTCAGACGTGT GCTCTTCCGATCT atggatgtggcgcaggta |
| RUNX1_1stF | tgaacccagcatagtggtca |
| RUNX1_1stR | aaagttctcacgcaccgact |
| RUNX1_2ndF | ACACTCTTTCCCTACACGAC GCTCTTCCGATCT ctagaggggtgaggctgaaa |
| RUNX1_2ndR | GTGACTGGAGTTCAGACGTGT GCTCTTCCGATCT ggtgaaacaagctgccattt |
| VEGFA_1stF | ttggtgccaaattcttctcc |
| VEGFA_1stR | agggagcaggaaagtgaggt |
| VEGFA_NGCG_2ndF | ACACTCTTTCCCTACACGAC GCTCTTCCGATCT gaagcaactccagtcccaaa |
| VEGFA_NGCG_2ndR | GTGACTGGAGTTCAGACGTGT GCTCTTCCGATCT cacacgcacacactcactca |
| VEGFA_NGA_1_2ndF | ACACTCTTTCCCTACACGAC GCTCTTCCGATCT aagcccattccctctttagc |
| VEGFA_NGA_1_2ndR | GTGACTGGAGTTCAGACGTGT GCTCTTCCGATCT attggaatcctggagtgacc |
| VEGFA_NGA_2_2ndF | ACACTCTTTCCCTACACGAC GCTCTTCCGATCT acagggaagctgggtgaat |
| VEGFA_NGA_2_2ndR | GTGACTGGAGTTCAGACGTGT GCTCTTCCGATCT gagagccgttccctctttg |
| NG-Cas9HC_g1_T7_FE | GAAATTAATACGACTCACTATAGcagaccggcggcttcagcaGTTTAAGAGCTATGCTGGAAAC |
| NG-Cas9HC_g2_T7_FE | GAAATTAATACGACTCACTATAGgtggcgtccagcacctcctGTTTAAGAGCTATGCTGGAAAC |
| NG-Cas9max_g1_T7_FE | GAAATTAATACGACTCACTATAGagaccgaggtgcagacaggGTTTAAGAGCTATGCTGGAAAC |
| NG-Cas9max_g2_T7_FE | GAAATTAATACGACTCACTATAGgtggcgtccagcacctcttGTTTAAGAGCTATGCTGGAAAC |
| EMX1_NGT_1stF | aaagcccatacatccacagc |
| EMX1_NGT_1stR | gactcttcctcccaccttcc |
| VEGFA_NGT_1stF | agctgtttgggaggtcagaa |
| VEGFA_NGT_1stR | gggataaaacccggatcaat |
| MECP2_NGT_1stF | catgcctggccaacttttat |
| MECP2_NGT_1stR | agccaaaagctaggcctctt |
| GRIN2B_1stF | ctttgtctggccttgctttc |
| GRIN2B_1stR | tctttgggtcggtctcattc |
| FANCF_NGA_1stF | acctggtgcagcaactcttt |
| FANCF_NGA_1stR | caggtctccagggcagttag |
| PTEN_1stF | gtcttttcaggcaggtgtca |
| PTEN_1stR | tacacccagcgattcctttc |
| SHNAK3_1stF | accatcctcaagtcgtccag |
| SHNAK3_1stR | ctgcagctctggagcaagtt |
| EMX1_NGT_2ndF | ACACTCTTTCCCTACACGAC GCTCTTCCGATCT ttcttcccacctggaatgtc |
| EMX1_NGT_2ndR | GTGACTGGAGTTCAGACGTGT GCTCTTCCGATCT gcactgactggacacaagtga |
| VEGFA_NGT_2ndF | ACACTCTTTCCCTACACGAC GCTCTTCCGATCT ggtcactccaggattccaatag |
| VEGFA_NGT_2ndR | GTGACTGGAGTTCAGACGTGT GCTCTTCCGATCT ccaaggttcacagcctgaaa |
| MECP2_NGT_2ndF | ACACTCTTTCCCTACACGAC GCTCTTCCGATCT aatggaagatccagagaaagc |
| MECP2_NGT_2ndR | GTGACTGGAGTTCAGACGTGT GCTCTTCCGATCT tgctactccagtgaacacacaa |
| GRIN2B_2ndF | ACACTCTTTCCCTACACGAC GCTCTTCCGATCT cctcctttgtctctgcctgt |
| GRIN2B_2ndR | GTGACTGGAGTTCAGACGTGT GCTCTTCCGATCT ggatctacatcacgtaacctgtctt |
| FANCF_NGA_2ndF | ACACTCTTTCCCTACACGAC GCTCTTCCGATCT ccgatgaggagacactccaa |
| FANCF_NGA_2ndR | GTGACTGGAGTTCAGACGTGT GCTCTTCCGATCT ccacaggctgctgagaaac |
| PTEN_2ndF | ACACTCTTTCCCTACACGAC GCTCTTCCGATCT agatttctaagccacagaaaaaga |
| PTEN_2ndR | GTGACTGGAGTTCAGACGTGT GCTCTTCCGATCT tggtgaccagcattttatgg |
| SHNAK3_2ndF | ACACTCTTTCCCTACACGAC GCTCTTCCGATCT ccgtccagctgcctgagc |
| SHNAK3_2ndR | GTGACTGGAGTTCAGACGTGT GCTCTTCCGATCT tcgagaatcccaatgtctcc |
